# Multiscale Modeling of Bone Healing: Toward a Systems Biology Approach

**DOI:** 10.3389/fphys.2017.00287

**Published:** 2017-05-08

**Authors:** Edoardo Borgiani, Georg N. Duda, Sara Checa

**Affiliations:** ^1^Julius Wolff Institute, Charité—Universitätsmedizin BerlinBerlin, Germany; ^2^Berlin-Brandenburg School for Regenerative Therapies, Charité—Universitätsmedizin BerlinBerlin, Germany

**Keywords:** bone healing, computer modeling, multiscale modeling, systems biology, tissue regeneration

## Abstract

Bone is a living part of the body that can, in most situations, heal itself after fracture. However, in some situations, healing may fail. Compromised conditions, such as large bone defects, aging, immuno-deficiency, or genetic disorders, might lead to delayed or non-unions. Treatment strategies for those conditions remain a clinical challenge, emphasizing the need to better understand the mechanisms behind endogenous bone regeneration. Bone healing is a complex process that involves the coordination of multiple events at different length and time scales. Computer models have been able to provide great insights into the interactions occurring within and across the different scales (organ, tissue, cellular, intracellular) using different modeling approaches [partial differential equations (PDEs), agent-based models, and finite element techniques]. In this review, we summarize the latest advances in computer models of bone healing with a focus on multiscale approaches and how they have contributed to understand the emergence of tissue formation patterns as a result of processes taking place at the lower length scales.

## Introduction

Bones have a number of different functions, but two of the most important ones are that bones provide structural support and physical protection of vital organs. Especially in elderly patients, bones can be fragile and frequently experience fractures. Although, bones have the fascinating and unique capacity to completely self-regenerate without leaving a scar; in some cases, such as in pathological fractures or those situations leading to large bone defects, bones fail to reach a complete healing. Delayed or incomplete bone regeneration represents a major clinical challenge. Nowadays, there is a need to understand the mechanisms involved in the bone healing process in order to develop treatment strategies which ensure the successful repair of the bone.

The unique process of bone healing is highly complex and dynamic and spans many different time and length scales. The process of healing can be divided into five overlapping phases: hematoma formation (pro-inflammation) phase, anti-inflammatory phase, soft callus formation (proliferative) phase, hard callus formation (maturing or modeling) phase, and remodeling phase (Schmidt-Bleek et al., 2014). During all these phases, many processes at the intracellular, cellular and tissue level scales are coordinated and interact to achieve bone restoration at the organ scale. At the intracellular level, a complex array of signaling molecules interacts and gives rise to the activation of specific genes that, ultimately, determine cell function. At the cellular level, cells proliferate, migrate, differentiate, and synthesize extracellular matrix. At the tissue level, bone, cartilage, and fibrous tissue are organized to provide the extracellular environment for the cells.

Over the last decades, experimental human and animal studies have provided detailed insights into the processes that occur during the bone healing response at individual length and time scales. For example, at the tissue level, it has been shown that bone healing is influenced by fixation stability (Schell et al., 2005), aging (Strube et al., 2008), fracture size (Claes et al., 1997), etc. At the cellular level, experimental studies have determined the contribution of the different cell phenotypes (immune cells, progenitor cells, etc.) to the healing response (Konnecke et al., 2014). At the intracellular level, the role of intracellular signaling pathways and their potential as therapeutic targets for bone regeneration have been investigated (Secreto et al., 2009). Despite great advances in the field at the individual length and time scales, the behavior of the process as a whole remains unclear.

The complexity involved in bone regeneration has brought computational modeling as a core discipline into regenerative research. In bone healing, such computer models aim to identify underlying rules driving bone regeneration cascades. First computer models of bone healing were focused on understanding how mechanical stability and/or loading influence the bone healing outcome. Using finite element techniques, those models aimed to determine the local mechanical signals (strains, pressure, fluid velocity, etc.) within the regenerating region and how they relate to the formation of different tissue phenotypes over the course of healing (Carter et al., 1988; Claes and Heigele, 1999). Although, they were a breakthrough in the field, they were mainly focused on the mechanical aspects of bone healing, ignoring or highly simplifying the biology of the process. As a result, they could not explain many of the experimental observations, such as non-healing in large bone defects or the effect of growth factor stimulation (Carlier et al., 2014; Ribeiro et al., 2015).

Over the years, computer models have progressively increased their complexity, by adding more and more biological details and giving rise to a new generation of computer models that try to understand mechanical and biological interactions occurring at the different time and length scales. One of the advantages of these more recent models lies in their ability not only to predict the bone healing outcome and how it might be influenced by, for example, fixation stability; but also to explain the mechanisms behind. Although mechanics can be used to predict the progression of bone healing in many situations, the mechanical conditions do not explain the bone healing process. Computer models that aim not only to predict but also to explain bone regeneration are based, at least to some extent, on the biological processes behind. Although some of these models have recently shown their potential to support the development of new treatment strategies for the promotion of bone repair (Carlier et al., 2014), most models have so far focused on understanding the mechanisms behind the bone healing response.

In what follows, we review how multiscale computer models have contributed to better understand the systems biology of the bone healing process. In the next section, we briefly describe the different modeling approaches that have been used to simulate bone regeneration. Thereafter, we group the models in three main categories based on the main biological processes under investigation: cellular activity (including migration, proliferation, differentiation, extracellular matrix production), growth factor production and effect, and angiogenesis.

## Modeling approaches

Modeling approaches in the field of bone regeneration have mostly focused on finite element, partial differential equations (PDEs) and agent-based techniques (Table 1). Finite element analyses have been used to solve the mechanical equations, i.e., to determine the mechanical signals within the regeneration region. Many models have then coupled these mechanical signals to changes in biological parameters; for example the differentiation or migration of the cells (Lacroix et al., 2002; Gomez-Benito et al., 2005; Isaksson et al., 2008; Nagel and Kelly, 2010; Checa et al., 2011). Following an iterative approach, these models simulate how changes in tissue patterning influence mechanical signals within the healing region and how these signals further influence tissue formation (Lacroix et al., 2002; Isaksson et al., 2006; Checa et al., 2011). Systems of PDEs have been used to simulate temporal and spatial changes in cell and tissue density or protein concentrations over the course of healing (Bailon-Plaza and van der Meulen, 2003; Gomez-Benito et al., 2005; Geris et al., 2010b). This modeling approach focuses on the processes occurring at the tissue and cell population (i.e., cell density) level. Models that take into account the processes at the cellular level are based on agent-based approaches. These models employ experimentally derived computational-coded rules to define the behavior of individual cells (Byrne et al., 2011; Checa et al., 2011). These models simulate the bone regeneration process at the cellular scale and aim to understand how cellular behavior gives rise to tissue formation pattern and bone regeneration.

**Table 1:** **Overview of the main properties of computer models of bone regeneration**.

**References**	**2D/3D**	**Scales considered**	**Modeling techniques**	**Connection between scales**			**Outcomes**
Carter et al., 1988	2D	Tissue level	Finite element modeling				Identification of intermittent hydrostatic stress as an important stimulus in the regulation of tissue patterning during bone healing
Claes and Heigele, 1999	2D	Tissue level	Finite element modeling				Quantification of the levels of mechanical strain leading to intramembranous bone formation, endochondral ossification, and fibrocartilage formation.
Lacroix et al., 2002	2D	Tissue level	Finite element modeling	Coupled equations			The site of origin of the cells has an important influence on the healing pattern.
		Cell population level	Partial differential equations				
Bailon-Plaza and van der Meulen, 2003	2D	Tissue level	Partial differential equations + finite element modeling	Coupled equations			Demonstrated the dependence of successful healing on moderate mechanical loading, and the adverse effects of insufficient, delayed, or excessive mechanical stimulation
		Cell population level	Partial differential equations				
Gomez-Benito et al., 2005	3D	Tissue level	Partial differential equations + finite element modeling	Coupled equations			Low fixation stiffness delays fracture healing and causes a larger callus
		Cell population level	Partial differential equations				
Geris et al., 2008	2D	Tissue level	Partial differential equations	Coupled equations			- The establishment of a vascular network in response to angiogenic growth factors as a key factor in the healing process. - A correct description of cell migration is essential to the prediction of realistic spatiotemporal tissue distribution patterns in the fracture callus.
		Cell population level	Partial differential equations				
Isaksson et al., 2008	2D	Tissue level	Partial differential equations + finite element modeling	Coupled equations			Identified matrix production rates of bone and cartilage, and cartilage replacement (degradation) as the most important parameters for the fracture healing process.
		Cell population level	Partial differential equations				
Nagel and Kelly, 2010	2D	Tissue level	Finite element modeling	Coupled equations			Collagen organization of the repair tissue is regulated by the local mechanical environment
		Cell population level	Partial differential equations				
Wehner et al., 2010	3D	Tissue level	Finite element modeling and fuzzy logic				Optimization of fixation stiffness for a reduced healing time.
Geris et al., 2010b	2D	Tissue level	Partial differential equations+ finite element modeling	Coupled equations			The direct action of mechanics on both angiogenesis and osteogenesis was able to predict overload-induced non-union formation
		Cell population level	Partial differential equations				
Checa et al., 2011	3D	Tissue level	Finite element modeling +agent-based model	Coupled equations			Inter-species differences in the mechanical regulation of bone healing between sheep and rat
		Cell level	Agent-based model				
Byrne et al., 2011	3D	Tissue level	Finite element modeling+ agent-based model	Coupled equations			Prediction of tissue differentiation patterns in an human tibia under realistic muscle loads
		Cell level	Agent-based model				
Vetter et al., 2012	2D	Tissue level	Finite element modeling	Coupled equations			Fracture bridging of the periosteal side by cartilage was observed only (i) for a specific choice of the mechanical threshold values for tissue differentiation and (ii) when assuming a strong source of biological stimulation at the periosteum
		Cell population level	Partial differential equations				
Carlier et al., 2012	2D	Tissue level	Partial differential equations	Coupled equations			- Increased tip cell density due to the loss of DII4 - Excessive number of tip cells in high VEGF environments - Absence of vascular network and fracture healing in very high VEGF environments
		Cell population level	Partial differential equations		Passing of variables		
		Cellular level	Agent-based model			Passing of variables	
		Intracellular level	Set of equations				
Steiner et al., 2013	3D	Tissue level	Finite element modeling and fuzzy logic				A fracture-healing model regulated by local distortional and dilatational strains was able to predict the course of IFM and tissue distribution of different healing situations under axial compression, torsion, shear loading, and bending.
Ribeiro et al., 2015	2D	Tissue level	Partial differential equations	Coupled equations			Bone healing in a large bone defect augmented with a BMP-2 soaked hydrogel as a result of the effect of BMP-2 on cellular activity
		Cell population level	Partial differential equations				

## The role of cellular activity on tissue formation patterns

Tissue patterning over the course of bone healing is an emergent event that results from multiple processes taking place at the lower length and time scales. For example, tissue patterning is determined by the number of cells present in the healing region, their location, migration, and proliferation capacity, as well as by the amount of extracellular matrix produced by the different cell phenotypes. Bailon-Plaza et al. were the first to explicitly introduce biological factors into a computer model of the bone healing process, including simulation of cell migration, proliferation and differentiation, as well as production and resorption of corresponding tissues (Bailon-Plaza and van der Meulen, 2001). Using PDEs, they determined spatial and temporal changes in the density of mesenchymal stem cells, osteoblasts and chondrocytes, as well as changes in bone and cartilage tissue densities inside the callus. Although, the model presented some major limitations, such as a highly simplified callus geometry, they were able to predict early periosteal bone formation and endochondral ossification in the external callus according to experimental observations. Thereafter, Bailon-Plaza et al. (Bailon-Plaza and van der Meulen, 2003) and others (Gomez-Benito et al., 2005; Isaksson et al., 2006, 2008; Garcia-Aznar et al., 2007; Geris et al., 2008, 2010a,b; Burke and Kelly, 2012) adapted and extended this model to investigate different aspects of the bone healing process, such as the effect of mechanical loading on the bone healing process. Bailon-Plaza et al. (Bailon-Plaza and van der Meulen, 2003) simulated the stimulatory or inhibitory effect of mechanical signals on the ossification process according to Claes et al. (Claes and Heigele, 1999). With this model, they were able to explain the beneficial and adverse effects of moderate and excessive loading, respectively, as well as the negative effects of delaying mechanical stimulation of rigidly fixed calluses; as observed experimentally. Geris et al. investigated the occurrence of non-unions due to mechanical over-loading (Geris et al., 2010b). In their model, they showed that the mechanical regulation of both angiogenesis and osteogenesis was able to predict overload-induced non-union, confirming the hypotheses of experimental studies investigating the interconnection between angiogenesis and osteogenesis. All these models based on PDEs make use of diffusion equations to simulate cellular invasion within the callus. Several models have used this approach to investigate the effect of the origin of the cells on the healing pattern (Lacroix et al., 2002; Vetter et al., 2012). Comparing model predictions with histological data, Vettel et al. showed that the source of progenitor cells needs to be stronger at the periosteum than at the marrow space in order to achieve early periosteal and delayed endosteal bone formation, as seen in bone healing experiments in sheep (Vetter et al., 2012).

Several computer models have used agent-based approaches to simulate the behavior of individual cells and to investigate their contribution to tissue patterning over the course of healing (Byrne et al., 2011; Checa et al., 2011; Carlier et al., 2012; Borgiani et al., 2015; O'Reilly et al., 2016). Using this approach, Checa et al. (2011) and Borgiani et al. (2015) showed that differences in the healing pattern (e.g., endosteal, periosteal, time to bringing) of different species (sheep, rat, and mice) can be explained by differences in the regulation of cellular activity by mechanical signals during the course of healing.

Statistical methods have been used to investigate the robustness of the models and to identify the influence of model parameters on model outcome. Using a design of experiments approach, Isaksson et al. determined the rate of bone tissue formation and cartilage degradation as key players for the prediction of uneventful bone fracture healing (Isaksson et al., 2008). Using the same approach, Carlier et al. identified the impaired endochondral ossification process and increased infiltration of fibroblastic cells as key contributors to the degree of severity of congenital pseudarthrosis of the tibia (Carlier et al., 2016).

## The role of intrinsic and extrinsic growth factors on cellular activity

Growth factors play a key role in the bone healing process by modulating cellular activity and serving as communication between the cells. A single growth factor may have effects on multiple cell types and induce different functions, resulting in a cascade of highly complex interactions. Several computer models have aimed to understand the role of growth factors on cellular activity, and thereof on tissue formation over the course of healing (Bailon-Plaza and van der Meulen, 2001; Moore et al., 2014; Ribeiro et al., 2015). Bailon-Plaza et al. assumed two initial sources of an osteogenic and a chondrogenic growth factor located at the periosteum and the hematoma, respectively; while keeping the duration of the release and the magnitude as parameters. They predicted that the duration of the release in the hematoma needs to be sufficiently long to initiate the healing response. They found an agreement with experimental data in the time point of peak osteogenic growth factor concentration (Bailon-Plaza and van der Meulen, 2001).

The chemotactic potential of vascular endothelial growth factors on the angiogenic process during bone healing has also been investigated using a computer model (Geris et al., 2008). This model suggested that endothelial cells are attracted toward gradients of vascular endothelial growth factor secreted by chondrocytes and osteoblast and that these gradients are ensured by the consumption of the growth factor by the endothelial cells. Based on these assumptions, the model was able to simulate compromised healing conditions due to impaired angiogenesis. However, the model did not take into account the influence of the mechanical environment on growth factor production or its effect on vascular growth.

Moore et al. explicitly included the effect of mechanics on the production of bone morphogenetic protein 2 (BMP-2) by progenitor cells located in the periosteum of a critical sized femoral defect in sheep stabilized with an intramedullary nail (Moore et al., 2014). Assuming that BMP-2 in turn regulates cell proliferation and differentiation, they were able to predict experimentally observed tissue formation over the course of healing.

The potential of growth factor BMP-2 incorporation to promote cellular activity and enhance tissue regeneration in the context of large bone defects has been shown not only in a clinic setting (Schmidt-Bleek et al., 2016), but also using computer modeling techniques (Ribeiro et al., 2015). Ribeiro et al. collected quantitative experimental data from the literature on the effect of BMP-2 on cellular activity (proliferation, migration, differentiation, extracellular matrix production, etc.) and implemented them into a model to simulate bone healing in a large defect stimulated with BMP-2 (Ribeiro et al., 2015). The authors showed a good agreement with experimental data on the amount of bone tissue formed over the course of healing; however they were not able to predict endosteal bone formation.

## Angiogenesis and the role of oxygen supply for successful healing

Angiogenesis, defined by the formation of new capillaries, plays a key role in bone repair. Several computational studies have been developed to investigate the relative role of revascularization on bone healing progression (Geris et al., 2008; Chen et al., 2009; Peiffer et al., 2011; Simon et al., 2011; Burke and Kelly, 2012; O'Reilly et al., 2016). These models have seen a clear evolution toward a multiscale approach with the aim to better reflect and understand the biology of the process. While early models simulated vascular growth as a simple diffusion process (Geris et al., 2008; Chen et al., 2009; Burke and Kelly, 2012), more recent models use a discrete approach in order to simulate the complex structure of the newly formed capillary network (Carlier et al., 2015; O'Reilly et al., 2016).

Diffusion based models have investigated the influence of angiogenic growth factor production or callus size on the establishment of the vascular network and in turn on successful bone healing (Geris et al., 2008; Chen et al., 2009). Computer models at the cellular level have focused on understanding how progenitor cell fate might be affected by the altered conditions present in fractures with disrupted vasculature (O'Reilly et al., 2016) or in genetically modified mouse animal models (Burke et al., 2013). The MOSAIC model, developed by Carlier and her group, is the only multiscale computer model of bone healing that incorporates processes at the intracellular scale (Carlier et al., 2012). It implements an intracellular module that includes Dll4/Notch1 signaling to determine tip cell selection. The model was able to simulate the salt and pepper pattern seen for cell fates, an increased tip cell density due to the loss of Dll4 and an excessive number of tip cells in high VEGF environments (Carlier et al., 2012).

## Discussion and future perspectives

Computer models can provide great insights into the complexity of the bone regeneration process, especially on the interactions between the different length and time scales. Despite significant advances in the field of computer modeling of bone healing and its clear trend toward a multiscale approach to provide a systems biology overview of the process, many limitations remain. In what follows, we describe some of the remaining challenges and give insights into possibilities for future work.

### Cells as “sensors” of the mechanical environment

So far, models have considered that the mechanical signals at the tissue level regulate the bone healing progression (e.g., Lacroix et al., 2002). However, it is well-known that, in reality, it is the cells that feel mechanical signals and respond with changes in cellular function (review in Su et al., 2011). We and others have shown that mechanical strains at the tissue level differ from those at the cellular scale (Bell et al., 2012; Shoham and Gefen, 2012; Checa et al., 2015). It is known that cells can sense mechanical stimuli provided by the surrounding matrix and that these stimuli influence cellular function, such as proliferation (Hadjipanayi et al., 2009), migration (Lo et al., 2000), and differentiation (Engler et al., 2006). However, the role of the mechanical regulation of cellular activity on processes at a larger scale, such as the formation/regeneration of bone, remains poorly understood.

**Figure 1:**
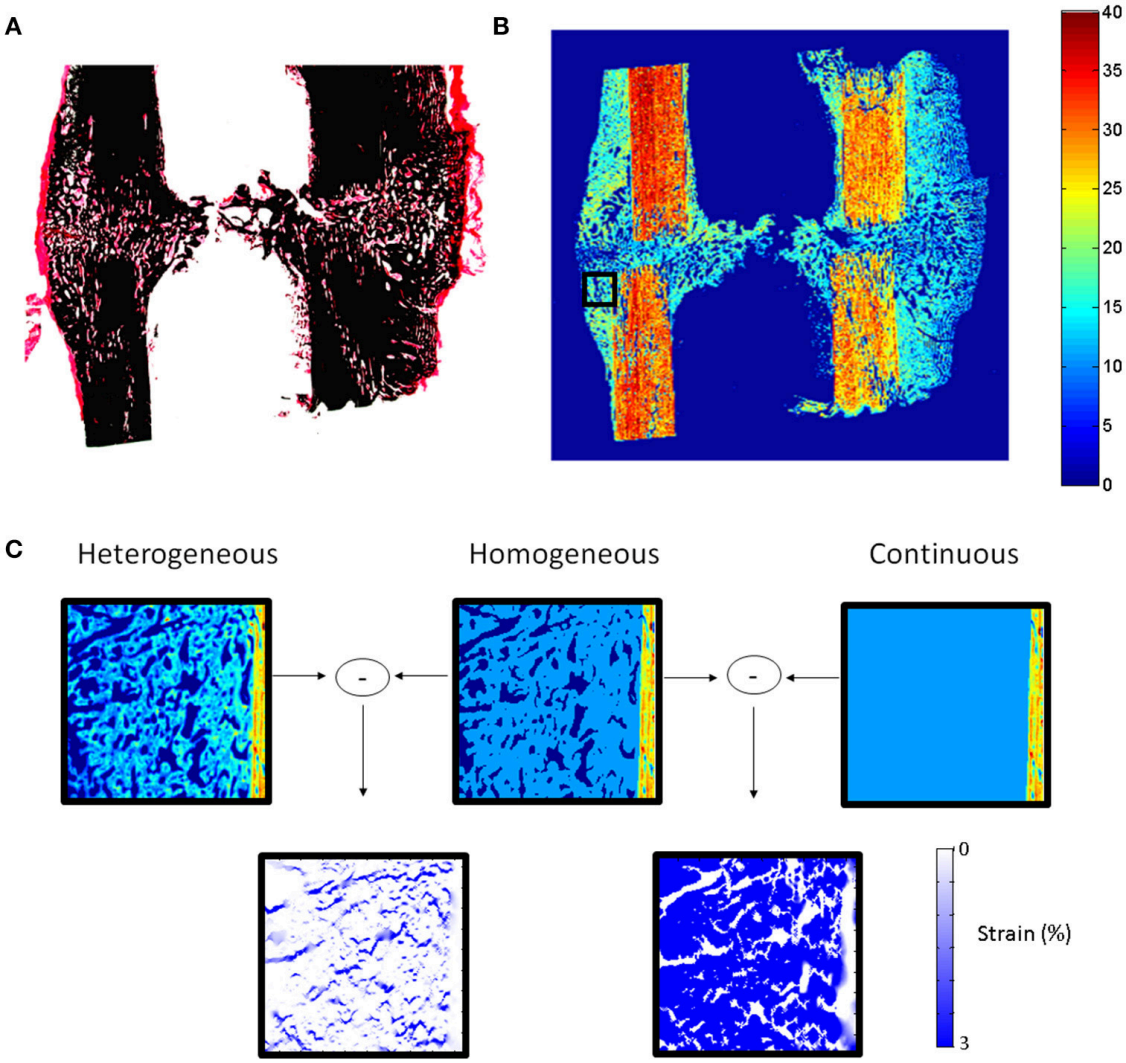
**(A)** Histological section (Safranin-O von Kossa staining) of sheep callus stabilized with an external fixator (9 weeks). **(B)** Map of elastic coefficient (GPa) of the same sample measured by quantitative acoustic scanning microscopy. **(C)** FEMs of callus region (black square **B**) under 10% compression showing the influence of callus tissue structure and heterogeneity on the mechanical strains within the healing region. High mechanical strains are induced in regions between the highly organized bone tissue, which cannot be predicted when describing the tissues as continuous and homogeneous materials.

Cells produce highly organized fibrous and mineralized structures with preferential orientations (Liu et al., 2010; Preininger et al., 2011), resulting in highly heterogeneous and anisotropic material behavior. One of the current limitations of computer models of bone healing is that they assume homogeneous distributions of tissues while, in reality, tissues within the healing region form very organized structures (Figure 1). A first step toward understanding the role of cellular mechanosensation on bone healing is to be able to determine how mechanical signals are transferred from the tissue to the cellular level. To achieve this, there is a need to develop realistic computer models of extracellular matrix production and organization.

### Early phases of healing: inflammation and the immune response

During the first phase of bone fracture healing, a hematoma is formed where platelets and macrophages are known to release different signaling molecules such as cytokines and growth factors. These molecules play a key role in the regulation of the subsequent cellular events during the healing process. More and more, it is becoming obvious that the initial healing and inflammation phases play a main role in the bone healing outcome and that understanding the mechano-biology of the initial stages of healing is important to improve bone fracture healing (Klein et al., 2003; Schlundt et al., 2015). Up to now, there are no computer models investigating the interactions occurring during this healing phase or considering the role of immune cells on bone regeneration. Future computer models of bone healing should aim at investigating the interactions that occur during the initial healing phases and how they influence healing progression.

### Reaching the intracellular level

**Figure 2:**
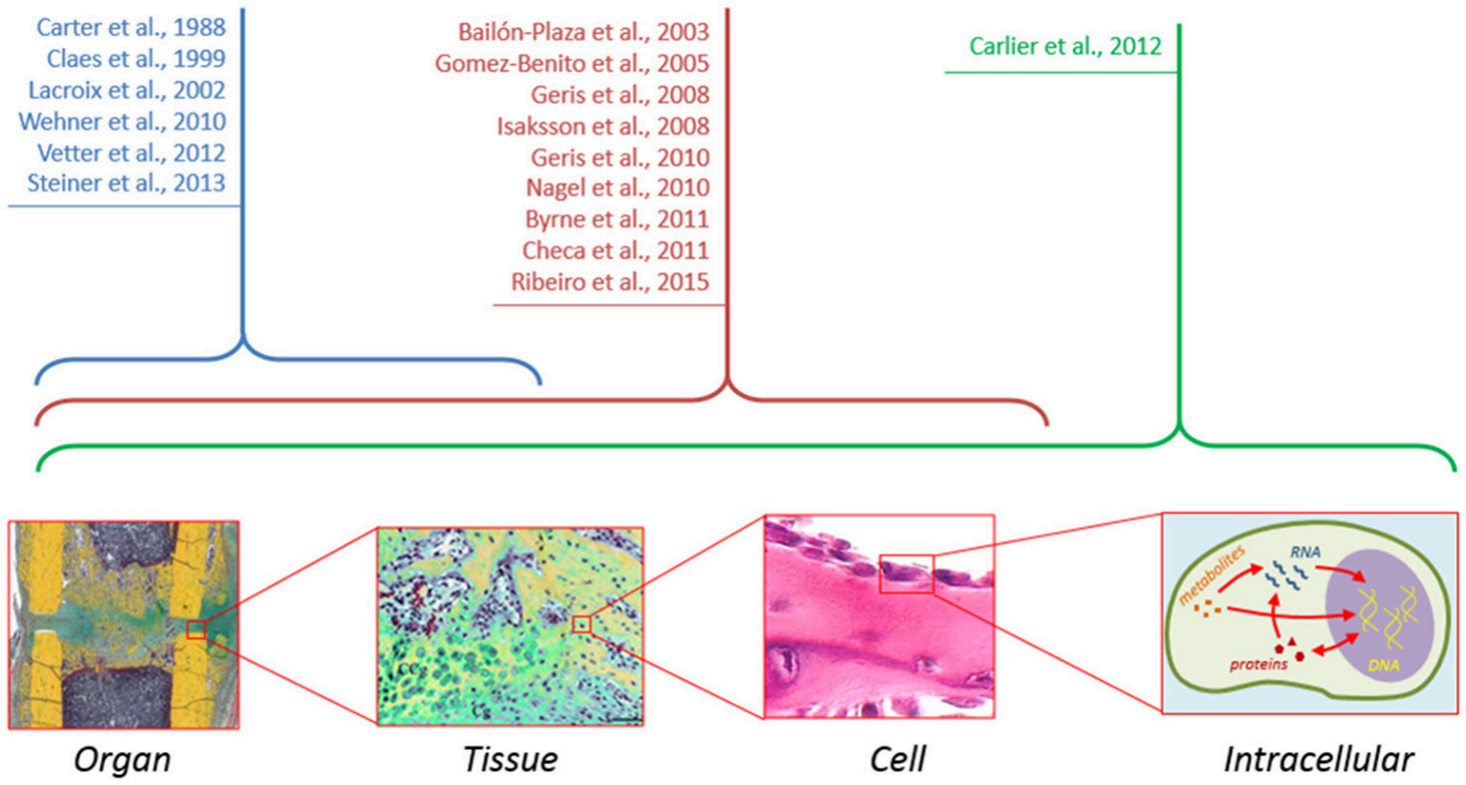
**Although computer models of bone healing tend toward a multiscale approach to understand interactions between and within the different length and time scales, computer models at the intracellular level are still lacking**.

Ultimately, cell decisions about differentiation, proliferation, and other cellular activities, are made on the basis of external signals. These signals are transmitted to the interior of the cells activating diverse signaling pathways which, in turn, activate genes involved in the specified cellular function. Several signaling pathways (Wnt, BMP, and ER receptor) have been shown to play a key role in the bone formation response (Hayrapetyan et al., 2015) and clinical strategies are being developed based on the modulation of these pathways to promote bone repair. However, experimentally, it is difficult to investigate the detailed mechanism of these pathways, their interactions and their implications at the cellular and tissue levels. Several studies have shown a great potential of computational approaches to elucidate non-obvious interactions between signaling pathways and their implications for cellular function (Gilbert et al., 2006). To date, the intracellular level has been mainly ignored in computer models of bone healing (Figure 2). Future *in silico* studies should aim at integrating information within and across all the different scales in order to get a mechanistic insight of the process as a whole.

### Modeling reusability and reproducibility

While computer modeling in systems biology has seen a strong advancement toward the development of model standards, reusability and reproducibility (Waltemath and Wolkenhauer, 2016), the field of computer modeling of bone regeneration lags clearly behind in this respect. To the author's knowledge, none of the computer models of bone regeneration developed so far has been made publically available in an open database. This, despite the fact that several journals, such as BMC, FEBS, or PLOS, request the authors to provide the model code through open repositories. Future developments in the field would definitely benefit from sharing, not only of model equations and parameters, as done so far, but also of model code and software. This would clearly facilitate the reproducibility of study results and the further development of the models.

## Conclusions

Despite advances in modeling being rather rapid, the present computer models are still unable to capture many of the mechanical and biological interactions that occur across and within the different time and length scales. More importantly, in most cases, they lack prediction power in compromised repair conditions. Old age (Bak and Andreassen, 1989), immune compromise (Claes et al., 2012) or genetic disorders (El Khassawna et al., 2012) have been shown to negatively affect the bone healing response; however little is known about the mechanisms behind these phenomena. From a computational point of view, very little has been done to understand how these factors influence the bone healing cascade. *In silico* studies show a great potential to contribute to the understanding of bone healing in compromised conditions and the consequences of alterations in cellular function on the bone healing outcome.

## Author contributions

EB and SC drafted the manuscript; all authors read and revised the manuscript and approved its content.

## Funding

This study was supported by the German Research Foundation (Deutsche Forschungsgemeinschaft: DU298/14-1; CH 1123/4-1).

### Conflict of interest statement

The authors declare that the research was conducted in the absence of any commercial or financial relationships that could be construed as a potential conflict of interest.
